# Supplementary material for: Adar RNA editing-dependent and -independent effects are required for brain and innate immune functions in Drosophila
Source: Nat Commun. 2020 Mar 27;11:1580. doi: 10.1038/s41467-020-15435-1 (PMC7101428; doi:10.1038/s41467-020-15435-1)
Supplement: Supplementary file 1 — Supplementary Information [file 41467_2020_15435_MOESM1_ESM.pdf]

SUPPLEMENTARY MATERIAL

Adar RNA editing in *Drosophila* is required for brain function and prevents antiviral Dicer-2 signaling

Patricia Deng[1*], Anzer Khan[2,3*], Dionna Jacobson[1], Nagraj Sambrani[2], Leeanne McGurk[4], Xianghua Li[4], Aswathy Jayasree[2], Jan Hejatko[2,3], Galit Shohat-Ophir[5], Mary A. O'Connell[2], Jin Billy Li[1#] and Liam P. Keegan[2#]

[1] Department of Genetics, Stanford University, Stanford, California, USA. [2] Central European Institute of Technology, Masaryk University, Brno, Czech Republic. [3] National Centre for Biomolecular Research, Faculty of Science, Masaryk University, Kamenice 5, 625 00 Brno, Czech Republic. [4] MRC Institute of Genetics and Molecular Medicine, Western General Hospital, Crewe Road, Edinburgh, EH4 2XU [5] The Faculty of Life Sciences and The Multidisciplinary Brain Research Center, Bar Ilan University, Ramat Gan, Israel

[*] Joint first author

# Joint corresponding authors

Correspondence should be addressed to L.P.K. (liam.keegan@ceitec.muni.cz) or J.B.L. (jin.billy.li@stanford.edu)

Running title: Inactive *Adar*

Key words: ADAR; RNA editing; *Drosophila*, neurodegeneration

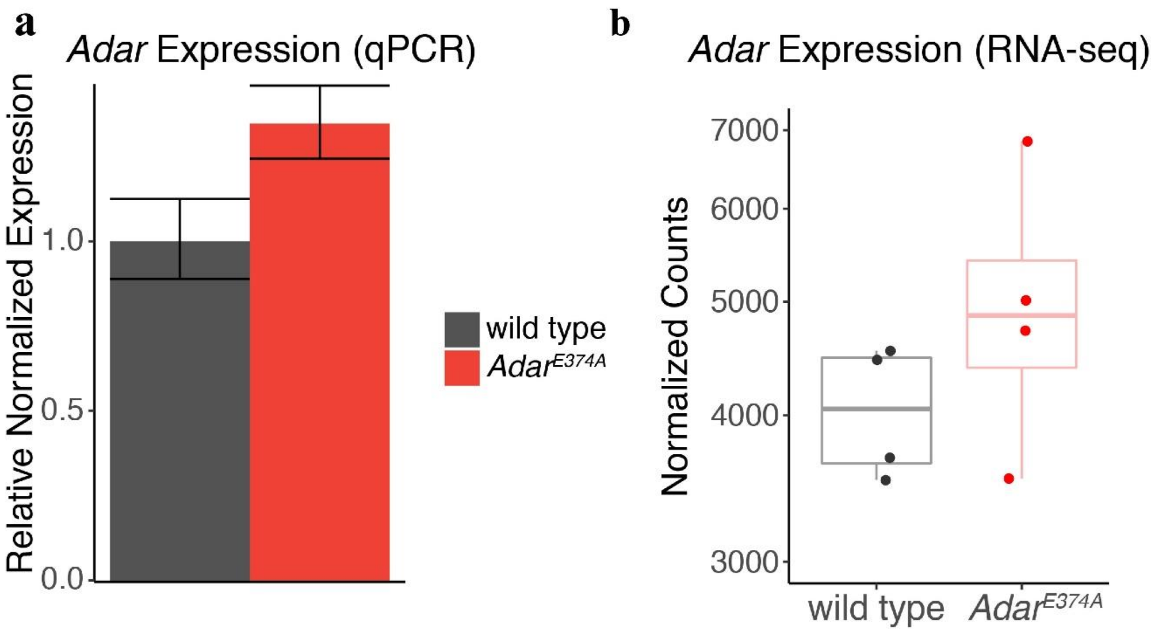

Supplementary Figure 1: Expression of Adar in *Adar*^{E374A} vs. wild type flies. (**a**) *Adar* gene expression from qPCR of 2-3 day old whole male flies. T-test p-value for *Adar*^{E374A} vs. wild type = 0.114. Error bars: SEM. (**b**) *Adar* gene expression from RNA-seq of 3 day old male fly heads (DESeq2). Boxplots are overlaid by points representing the biological replicates. The y-axis uses a log scale. (DESeq2 Wald test p-value = 0.105219 (Benjamini & Hochberg adjusted p-value = 0.5721788))

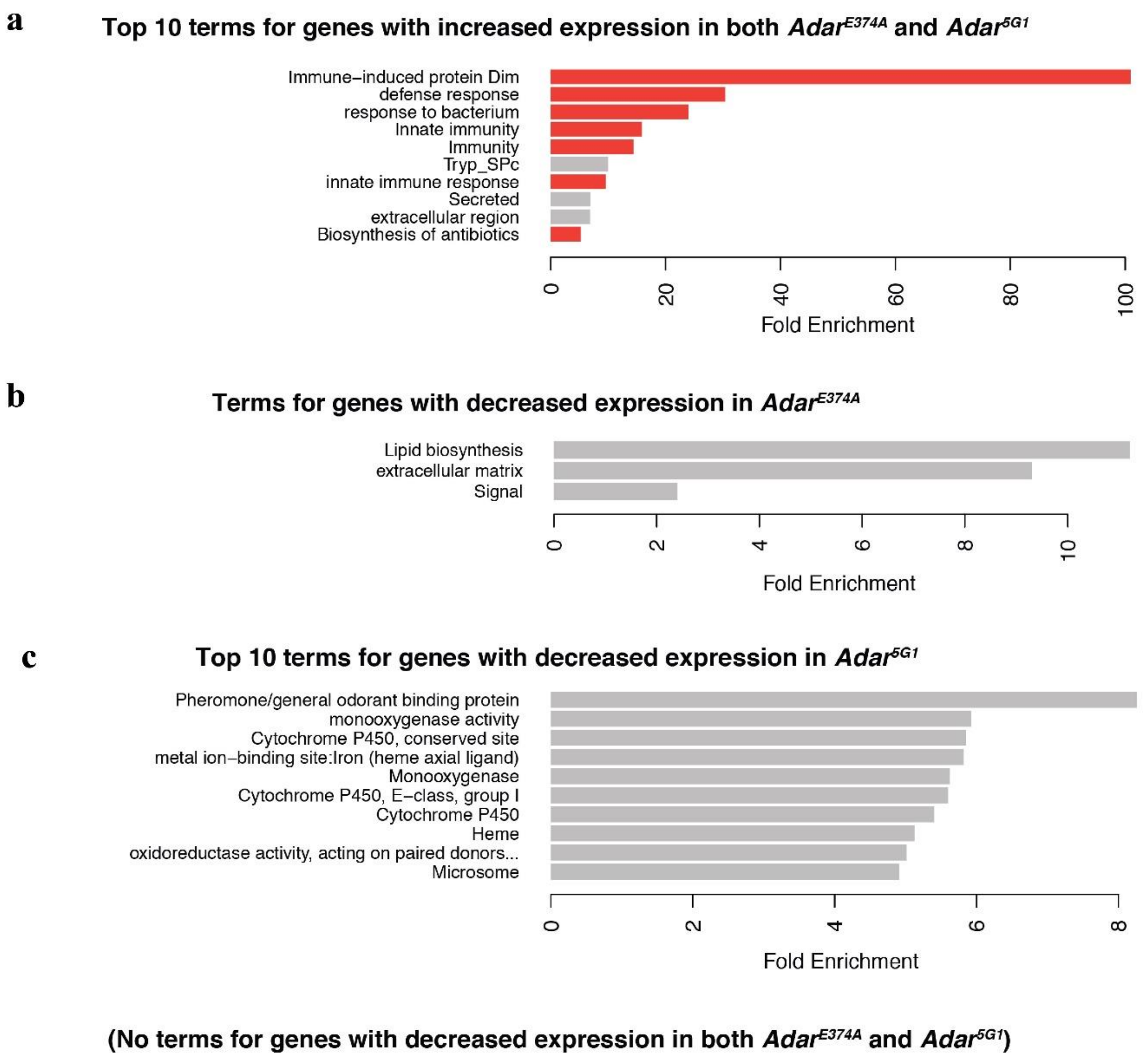

Supplementary Figure 2: GO term enrichment for differentially expressed genes in *Adar* mutant RNAseq. Bars are colored red for immune-related terms. The terms used in the analysis are the DAVID default terms (biological process, molecular function, and cellular component GO terms, clusters of orthologous groups (COG), UniProt keywords and sequence features, Kyoto Encyclopedia of Genes and Genomes (KEGG) pathways, InterPro, Protein Information Resource (PIR) superfamily, and SMART). (**a**) The top 10 out of 15 significantly enriched terms for the genes with increased expression in both the *Adar*^{E374A} and *Adar*^{5G1} mutants. (DESeq2: 10% FDR; DAVID: $\log_2$FoldChange >= 0.6, Benjamini adjusted p-value < 0.01) (**b**) The significantly enriched terms for the genes with decreased expression in the *Adar*^{E374A} mutant. (Same analysis parameters as in (**a**).) (**c**) The top 10 out of 17 significantly enriched terms for the genes with decreased expression in the *Adar*^{5G1} mutant. (Same analysis parameters as in (**a**)) There were no significantly enriched terms for genes with decreased expression in both the *Adar*^{E374A} and *Adar*^{5G1} mutants.

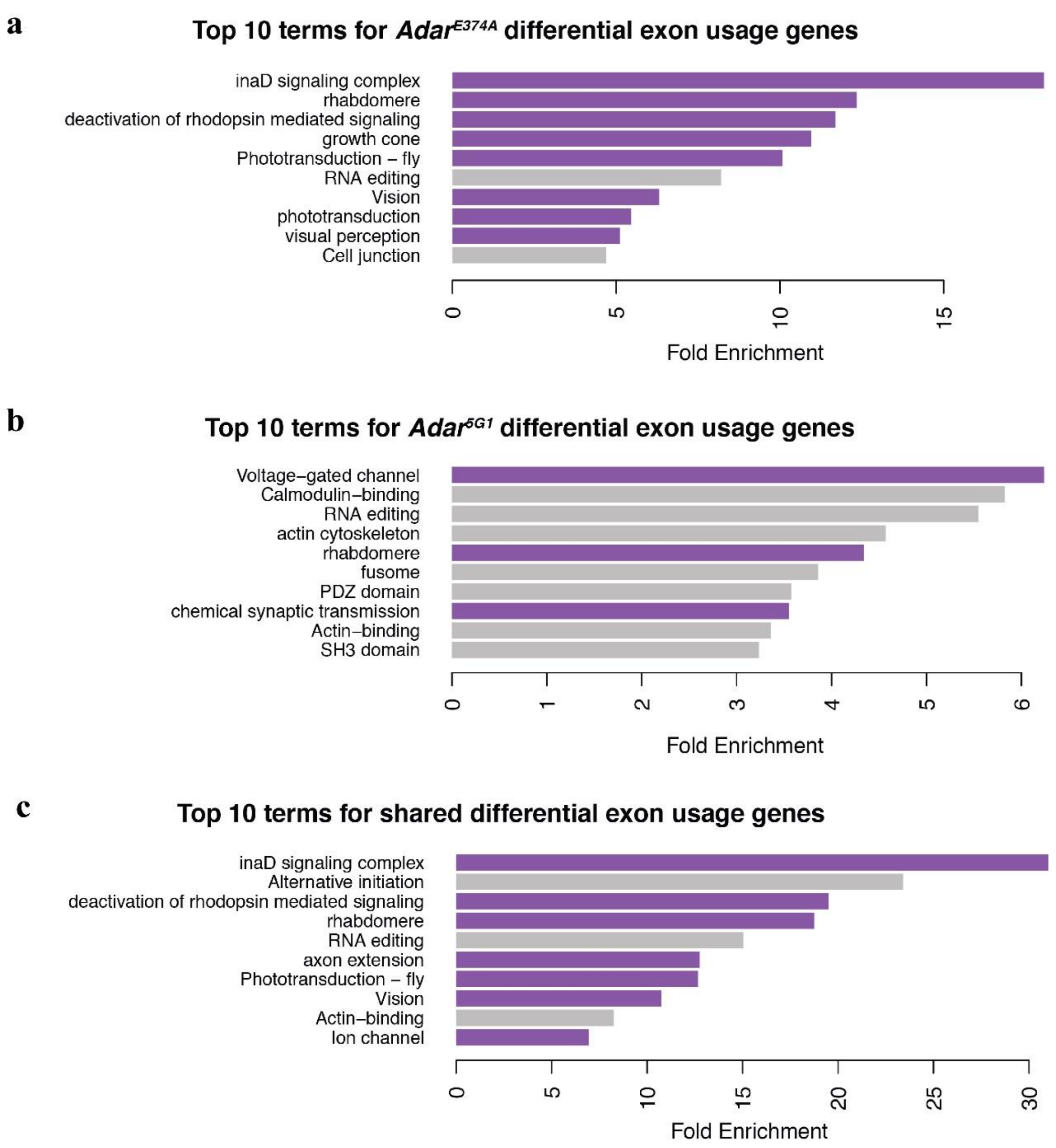

Supplementary Figure 3: GO term enrichment for genes with differential exon usage in *Adar* mutant RNAseq. Bars are colored purple for neurological-related terms. The terms used in the analysis are the DAVID default terms (biological process, molecular function, and cellular component GO terms, clusters of orthologous groups (COG), UniProt keywords and sequence features, Kyoto Encyclopedia of Genes and Genomes (KEGG) pathways, InterPro, Protein Information Resource (PIR) superfamily, and SMART). (**a**) The top 10 out of 21 significantly enriched terms for genes with differential exon usage in the *Adar*^{E374A} mutant. (DEXSeq: 10% FDR; DAVID analysis Benjamini adjusted p-value < 0.01) (**b**) The top 10 out of 35 significantly enriched terms for the genes with differential exon usage in the *Adar*^{5G1} mutant. (**c**) The top 10 out of 22 significantly enriched terms for the genes with differential exon usage in both the *Adar*^{E374A} and *Adar*^{5G1} mutants. (Same analysis parameters as in (**a**))

a

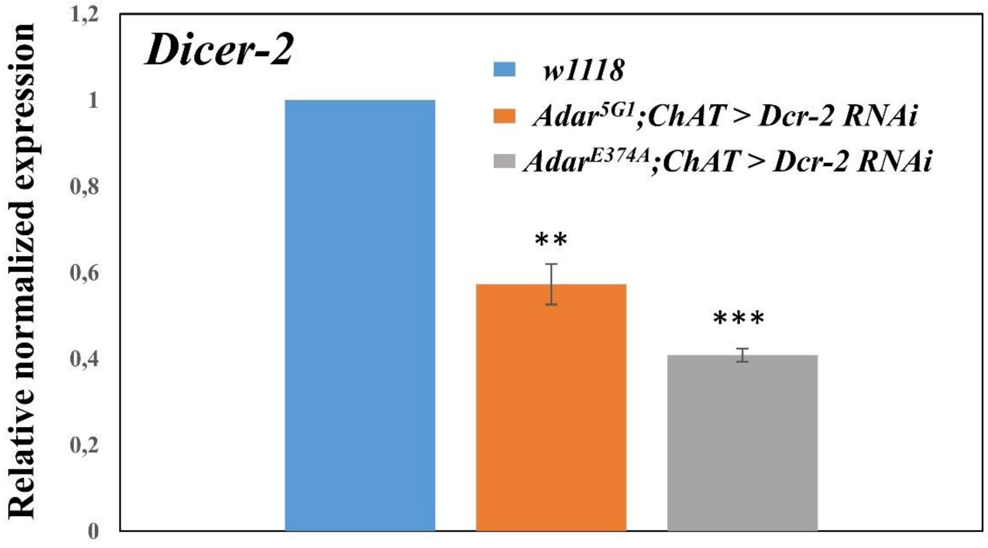

b

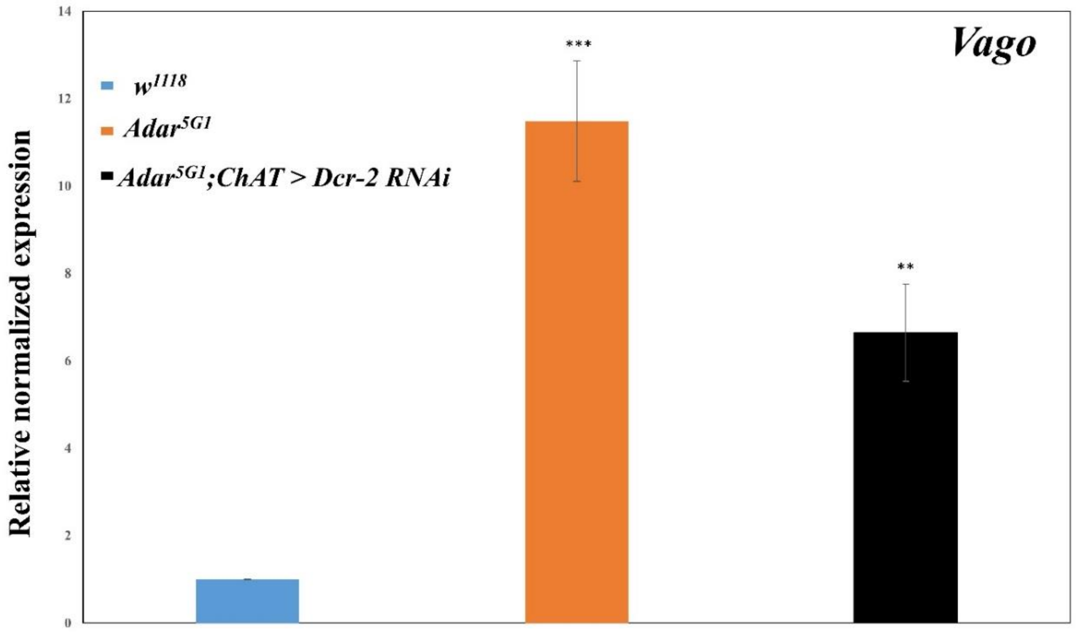

Supplementary Figure 4: Knocking down *Dicer-2* by RNAi in cholinergic neurons rescues the *Vago* expression partially. a. qRT-PCR of *Dcr-2* transcripts in *Dcr-2* knockdown shows that the *Dcr-2* transcript is reduced compared to the wild type **b.** qRT-PCR of *Vago* transcripts in *Adar*^{5G1} null mutant and in *Adar*$^{5G1;}$ *ChAT > Dcr-2* RNAi shows that the aberrant *Vago* induction in the *Adar*^{5G1} is partially rescued by knocking down *Dcr-2*. *p*-values were calculated by Stdents t-test ***:** p-value < 0.05. **: p-value < 0.01. ***: p-value < 0.005.: n.s – not significant. Error bars: SEM (Standard Error of Mean for biological replicates).

a

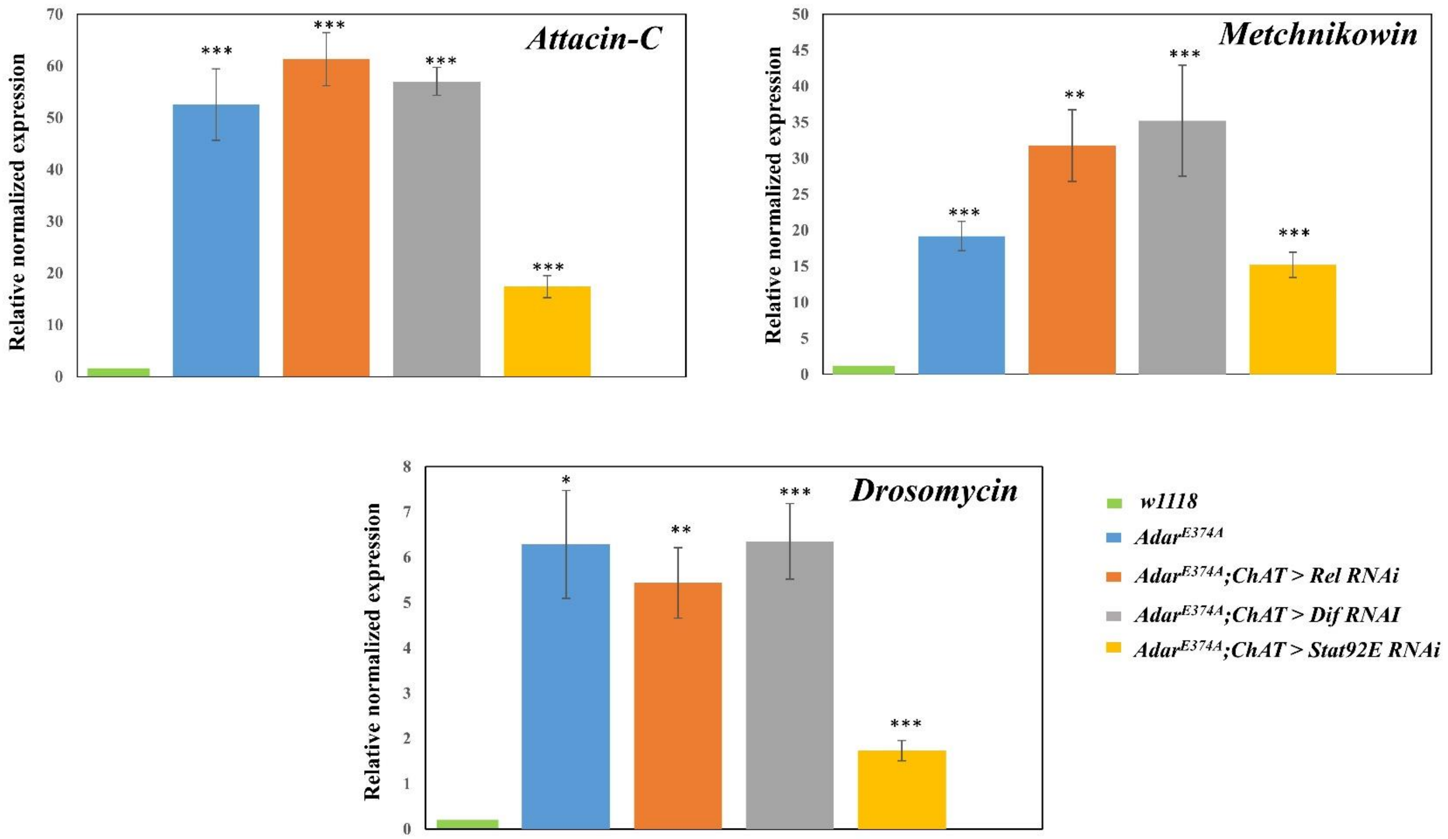

b

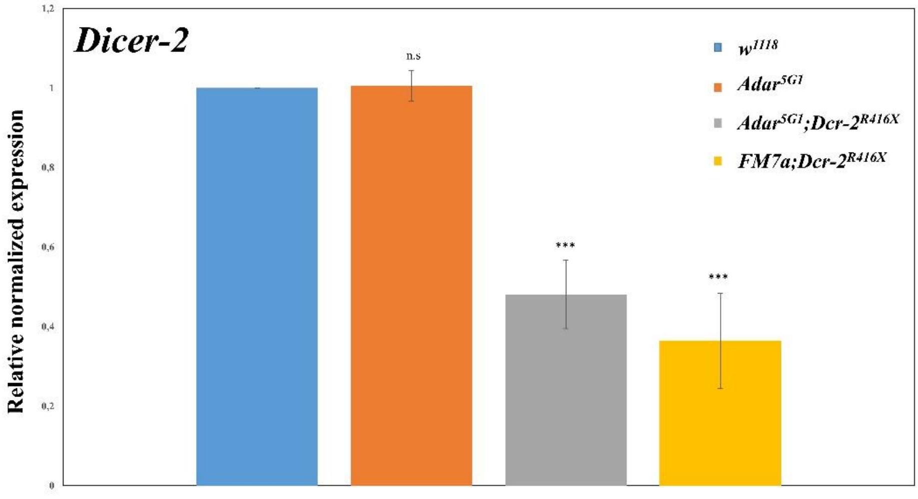

Supplementary Figure 5: Aberrant innate immune induction in the *Adar*^{E374A} mutant also involves Jak-Stat signaling pathways. a Aberrant expression of AMP transcripts in head RNA of *Adar*^{E374A}*; ChAT>Rel RNAi*, *Adar*^{E374A}*; ChAT>Dif RNAi* or *Adar*^{E374A}*; ChAT>Stat92E RNAi* flies with RNAi knockdowns of *Rel* , *Dif* and *Stat92E* transcripts respectively , **b.** qRT-PCR of *Dcr-2* transcripts in *Dcr-2*^{R416X} double mutants shows that the *Dcr-2* transcript is expressed. Levels of each transcript in each of the different fly head RNA samples is compared to that in *w*^{1118}**.** *p*-values were calculated by Stdents t-test ***:** p-value <

0.05. **: p-value < 0.01. ***: p-value < 0.005.: n.s – not significant. Error bars: SEM (Standard Error of Mean for biological replicates).

Supplementary Table 1: qPCR Primers

**Name**	**Sequence**	**Note**
RP49_F	CCGCTTCAAGGGACAGTATC	
RP49_R	GACAATCTCCTTGCGCTTCT	
diptericin_F	ACCGCAGTACCCACTCAATC	
diptericin_R	ACTTTCCAGCTCGGTTCTGA	
IM3_F	ACTCGCCTTCGTTTTGGGTC	
IM3_R	TTAGGCCCTCACATTGCAGA	
IM1_F	TGCCCAGTGCACTCAGTATC	
IM1_R	GATCACATTTCCTGGATCGG	
IM2_F	AAATACTGCAATGTGCACGG	
IM2_R	ATGGTGCTTTGGATTTGAGG	
CanA1_F	CCGCCAGTGGAAACAAACAG	From FlyPrimerBank
CanA1_R	GCGGAAGTGGAACATCATCG	
IM2_F	AAATACTGCAATGTGCACGG	
IM2_R	ATGGTGCTTTGGATTTGAGG	
CG16836_F	TCAAGTCGCCGGAACTTTGA	
CG16836_R	ATACACTTGTCCCGGCGTAG	
CG15067_F	GAGCCTGACGTTATTGGCG	
CG15067_R	CCTTTTCCACTTGTTGGCTTGT	
CG15068_F	TTTCTCTTCGGACTTTTGGCTT	
CG15068_R	AAACTTTGCAGTCGCCGTTTA	
Adar_F	GGCTATAACCGAAAATTGCCACA	
Adar_R	TGTCTTAGCTCATTCAGCATGG	
Drosomycin_F	CTCCGTGAGAACCTTTTCCA	
Drosomycin_R	ACAGGTCTCGTTGTCCCAGA	
TotC_F	TACTATGCCTTGCCCTGCTC	
TotC_R	CAGATTCCCTTTCCTCGTCA	
Pp2B-14D_F	TGGACATGGGTAAGCTGTGC	
Pp2B-14D_R	CCAATTCAGACCTCGCCAGA	
CanA-14F_F	TGGATGCCGTCAACGAACG	
CanA-14F_R	CTGACAGTGATTTCTGGCCCTC	
CG18107_F	TGGCCAATGCTATTCCGTTGT	
CG18107_R	TTGCAATTCACACAGTCGCC	
IM23_F	GCTACGGCCGGAAATGTGA	
IM23_R	GTCCTGTAGGATTGGCCACC	
Metchnikowin_F	TACATCAGTGCTGGCAGAGC	
Metchnikowin_R	AATAAATTGGACCCGGTCTTG	
Vago_F	CAGCCAAGCGATTCCTTATC	
Vago_R	CTCATACAGTGGGCAGCATC	
Attacin-c_F	TTGGGTGGATCACTCACATC	
Attacin-c_R	GCGTATGGGTTTTGGTCAGT	

Def_F	GCTATCGCTTTTGCTCTGCT	
Def_R	GGTGTGGTTCCAGTTCCACT	
Dcr-2_F	GCTTTTATGTGGGTGAACAGGG	
DCR-2_R	GGCTGTGCCAACAAGAACTT	
Attacin-D_F	AGTTTATGGAGCGGTCAACG	
Attacin-D_R	CGATCGGCTATGACTGTGAA	
Tot A_F	TTCAGCGTTCCAAAAAGTCA	
Tot A_R	CGATACTCTCCCGTTCCTCA	
Tot X_F	GCAGACAGGCAACAATTTGA	
Tot X_R	TATACCGGGTTCCGACTCTG	
CG15065_F	ATGAAGTGGATGTCCTTGGTCT	
CG15065_R	CATTACCCGGATTTAACGGAGAA	
