# Supplementary material for: Adar RNA editing-dependent and -independent effects are required for brain and innate immune functions in Drosophila
Source: Nat Commun. 2020 Mar 27;11:1580. doi: 10.1038/s41467-020-15435-1 (PMC7101428; doi:10.1038/s41467-020-15435-1)
Supplement: Supplementary file 3 — Reporting Summary [file 41467_2020_15435_MOESM3_ESM.pdf]

nature research

Corresponding author(s): Liam Keegan

Last updated by author(s): Nov 8, 2019

Reporting Summary

Nature Research wishes to improve the reproducibility of the work that we publish. This form provides structure for consistency and transparency in reporting. For further information on Nature Research policies, see Authors & Referees and the Editorial Policy Checklist.

Statistics

For all statistical analyses, confirm that the following items are present in the figure legend, table legend, main text, or Methods section.

n/a	Confirmed	
☐	☒	The exact sample size (*n*) for each experimental group/condition, given as a discrete number and unit of measurement
☐	☒	A statement on whether measurements were taken from distinct samples or whether the same sample was measured repeatedly
☐	☒	The statistical test(s) used AND whether they are one- or two-sided<br>*Only common tests should be described solely by name; describe more complex techniques in the Methods section.*
☒	☐	A description of all covariates tested
☐	☐	A description of any assumptions or corrections, such as tests of normality and adjustment for multiple comparisons
☐	☒	A full description of the statistical parameters including central tendency (e.g. means) or other basic estimates (e.g. regression coefficient) AND variation (e.g. standard deviation) or associated estimates of uncertainty (e.g. confidence intervals)
☐	☒	For null hypothesis testing, the test statistic (e.g. *F*, *t*, *r*) with confidence intervals, effect sizes, degrees of freedom and *P* value noted<br>*Give P values as exact values whenever suitable.*
☒	☐	For Bayesian analysis, information on the choice of priors and Markov chain Monte Carlo settings
☒	☐	For hierarchical and complex designs, identification of the appropriate level for tests and full reporting of outcomes
☒	☐	Estimates of effect sizes (e.g. Cohen's *d*, Pearson's *r*), indicating how they were calculated

Our web collection on statistics for biologists contains articles on many of the points above.

Software and code

Policy information about availability of computer code

Data collection	Bio-Rad CFX Manager
Data analysis	DESeq2, DEXSeq, R, DAVID

For manuscripts utilizing custom algorithms or software that are central to the research but not yet described in published literature, software must be made available to editors/reviewers. We strongly encourage code deposition in a community repository (e.g. GitHub). See the Nature Research guidelines for submitting code & software for further information.

Data

Policy information about availability of data

All manuscripts must include a data availability statement. This statement should provide the following information, where applicable:

- Accession codes, unique identifiers, or web links for publicly available datasets
- A list of figures that have associated raw data
- A description of any restrictions on data availability

RNAseq data is available online at NCBI's GEO under GSE86054.

Field-specific reporting

Please select the one below that is the best fit for your research. If you are not sure, read the appropriate sections before making your selection.

☒ Life sciences ☐ Behavioural & social sciences ☐ Ecological, evolutionary & environmental sciences

For a reference copy of the document with all sections, see nature.com/documents/nr-reporting-summary-flat.pdf

Life sciences study design

All studies must disclose on these points even when the disclosure is negative.

Sample size	The sample size was always equal to or greater than 5 for behavioral studies .For RNA and Protein Isolation minimum 20 flies were taken and pool together to make one sample.
Data exclusions	No data were excluded from the analyses
Replication	All data presented here are representative of at least two independent experiments and all attempts of replication was successful.
Randomization	The experimental groups were allocated based on genotypes. Appropriate control groups were assayed in parallel.
Blinding	The investigators were not blinded during data collection or analysis.

Reporting for specific materials, systems and methods

We require information from authors about some types of materials, experimental systems and methods used in many studies. Here, indicate whether each material, system or method listed is relevant to your study. If you are not sure if a list item applies to your research, read the appropriate section before selecting a response.

Materials & experimental systems

n/a	Involved in the study	
☐	☒	Antibodies
☒	☐	Eukaryotic cell lines
☒	☐	Palaeontology
☐	☒	Animals and other organisms
☒	☐	Human research participants
☒	☐	Clinical data

Methods

n/a	Involved in the study	
☒	☐	ChIP-seq
☒	☐	Flow cytometry
☒	☐	MRI-based neuroimaging

Antibodies

Antibodies used	Anti-ADAR2 (HPA018277)<br>anti-Tubulin (Developmental Studies Hybridoma Bank)
Validation	Anti-ADAR2 (HPA018277)(Commercially available , Validated by the manufacturer extensively)<br>anti-Tubulin (Developmental Studies Hybridoma Bank) (Commercially available and deposited by a researcher and extensively used and validated by researchers all over world.

Animals and other organisms

Policy information about studies involving animals; ARRIVE guidelines recommended for reporting animal research

Laboratory animals	Drosophila melanogaster.
Wild animals	No wild animals were used.
Field-collected samples	This study does not involve samples collection from the field.
Ethics oversight	No ethical oversight was required as no vertebrate animals were involved in this study.

Note that full information on the approval of the study protocol must also be provided in the manuscript.
